# Midazolam’s Effects on Delayed-Rectifier K^+^ Current and Intermediate-Conductance Ca^2+^-Activated K^+^ Channel in Jurkat T-lymphocytes

**DOI:** 10.3390/ijms22137198

**Published:** 2021-07-04

**Authors:** Ning-Ping Foo, Yu-Fan Liu, Ping-Ching Wu, Chung-Hsi Hsing, Bu-Miin Huang, Edmund-Cheung So

**Affiliations:** 1 Department of Emergency Medicine, An Nan Hospital, China Medical University, Tainan City 709204, Taiwan; ningping.tw@gmail.com; 2 Graduate Institute of Medical Sciences, Chang Jung Christian University, Tainan City 711301, Taiwan; 3 Graduate Institute of Oncology, College of Medicine, National Taiwan University, Taipei City 100233, Taiwan; uys1222@gmail.com; 4 Department of Biomedical Engineering, National Cheng Kung University, Tainan City 70101, Taiwan; wbcxyz@gmail.com; 5 Institute of Oral Medicine and Department of Stomatology, National Cheng Kung University Hospital, College of Medicine, National Cheng Kung University, Tainan City 704302, Taiwan; 6 Center of Applied Nanomedicine, National Cheng Kung University, Tainan City 70101, Taiwan; 7 Medical Device Innovation Center, Taiwan Innovation Center of Medical Devices and Technology, National Cheng Kung University Hospital, National Cheng Kung University, Tainan City 704302, Taiwan; 8 Department of Anesthesia, Chi Mei Medical Center, Tainan City 710402, Taiwan; biohsing@gmail.com; 9 Department of Cell Biology and Anatomy, National Cheng Kung University, Tainan City 70101, Taiwan; 10 Department of Medical Research, China Medical University Hospital, China Medical University, Taichung 404332, Taiwan; 11 Department of Anesthesia & Medical Research, An Nan Hospital, China Medical University, Tainan City 709204, Taiwan

**Keywords:** midazolam, lymphocyte, delayed-rectifier K^+^ current, inactivation kinetics, intermediate-conductance Ca^2+^-activated K^+^ channel, interleukin

## Abstract

Midazolam (MDZ) could affect lymphocyte immune functions. However, the influence of MDZ on cell’s K^+^ currents has never been investigated. Thus, in the present study, the effects of MDZ on Jurkat T lymphocytes were studied using the patch-clamp technique. Results showed that MDZ suppressed the amplitude of delayed-rectifier K^+^ current (*I*_K(DR)_) in concentration-, time-, and state-dependent manners. The IC_50_ for MDZ-mediated reduction of *I*_K(DR)_ density was 5.87 μM. Increasing MDZ concentration raised the rate of current-density inactivation and its inhibitory action on *I*_K(DR)_ density was estimated with a dissociation constant of 5.14 μM. In addition, the inactivation curve of *I*_K(DR)_ associated with MDZ was shifted to a hyperpolarized potential with no change on the slope factor. MDZ-induced inhibition of *I*_K(DR)_ was not reversed by flumazenil. In addition, the activity of intermediate-conductance Ca^2+^-activated K^+^ (IK_Ca_) channels was suppressed by MDZ. Furthermore, inhibition by MDZ on both *I*_K(DR)_ and IK_Ca_-channel activity appeared to be independent from GABAA receptors and affected immune-regulating cytokine expression in LPS/PMA-treated human T lymphocytes. In conclusion, MDZ suppressed current density of *I*_K(DR)_ in concentration-, time-, and state-dependent manners in Jurkat T-lymphocytes and affected immune-regulating cytokine expression in LPS/PMA-treated human T lymphocytes.

## 1. Introduction

Midazolam (MDZ) is a common sedative drug for patients during procedures and surgery. It belongs to a group known as benzodiazepines, which are known to act on the central nervous system (CNS). In addition, being a CNS sedative drug, there are growing reports showing that this compound could influence mammalian immune function both in cell culture and in vivo [1,2,3,4,5,6,7,8,9]. More interestingly, it has been demonstrated to activate the intrinsic pathways of apoptotic changes through elicitation of the mitochondrial pathway [10,11].

Earlier reports showed that MDZ could be a direct regulator of ion channels, especially K^+^ channels, and that these actions are thought to have pharmacological, toxicological or immunological implications [12,13,14,15,16]. In particular, K_V_1.3 and Kv1.5-encoded currents, which exhibit unique gating properties and voltage dependency, largely constitute the delayed-rectifier K^+^ currents (*I*_K(DR_) in immune cells [3,16,17,18]. These currents, which can be functionally expressed in the plasma membrane and the inner mitochondrial membrane, have been demonstrated to perform key functions in the immune system, such as lymphocyte activation [3,15,18,19,20,21,22,23,24].

The intermediate-conductance Ca^2+^-activated K^+^ (IK_Ca_) channels (also known as K_Ca_3.1) are encoded by the *KCNN4* gene and strongly associated with various cellular functions, including the reciprocal regulation of Ca^2+^ influx and/or K^+^ efflux, as well as delicate control of cell growth and migration [25,26,27,28,29,30,31]. These channels have single-channel conductance of 20–60 pS and they are sensitive to be blocked by TRAM-34 and to be stimulated by DCEBIO [28,30,31]. Earlier reports also showed that the activity of IK_Ca_ channels in immune cells is important in influencing functional activities of these cells [20,27,28,32,33]. The modulators of IK_Ca_ channels have also been demonstrated to perturb immune functions [3,27,28,31,33,34,35]. However, whether MDZ and its structurally related compounds influence the activity of these channels in immune cells is largely unclear.

The Jurkat T cell line, a CD45-deficient clone derived from the E6-1 clone of Jurkat human T-cell leukemic cell line, has been demonstrated to express K_V_1.3-type *I*_K(DR)_ [15,17,18,24]. This cell line is recognized as a suitable model for investigations of lymphocytic functions, such as cellular activation and apoptosis [19,24]. Importantly, Jurkat T-lymphocytes have been demonstrated the presence of K_V_1.3 channels in the inner mitochondrial membrane, which is strongly linked to lymphocyte apoptosis [19,23,36,37]. An earlier report also showed the presence of peripheral-type benzodiazepine receptors (i.e., GABA_A_ receptors) in immune cells [38]. Whether MDZ can interact with GABA_A_ receptors to influence ion-channel function in lymphocytes remains to be studied. 

This study aimed to evaluate the effects of MDZ on ionic currents in Jurkat T-lymphocytes and in phytohemagglutinin (PHA)-preactivated human T lymphocytes, particularly at *I*_K(DR)_ and IK_Ca_ channels. We found that MDZ not only diminished the peak density of *I*_K(DR)_ (K_V_1.3-encoded current) but also shortened the time course of current inactivation. Additionally, this agent was also effective in suppressing the activity of IK_Ca_ channels in Jurkat T-lymphocytes. Therefore, it is anticipated that both inhibition of *I*_K(DR)_ and IK_Ca_-channel activity by MDZ may synergistically act to affect different functional activities of lymphocytes in cell culture and in vivo.

## 2. Results

### 2.1. Conjugation of MDZ with Jurkat Cell

**Figure 1:**
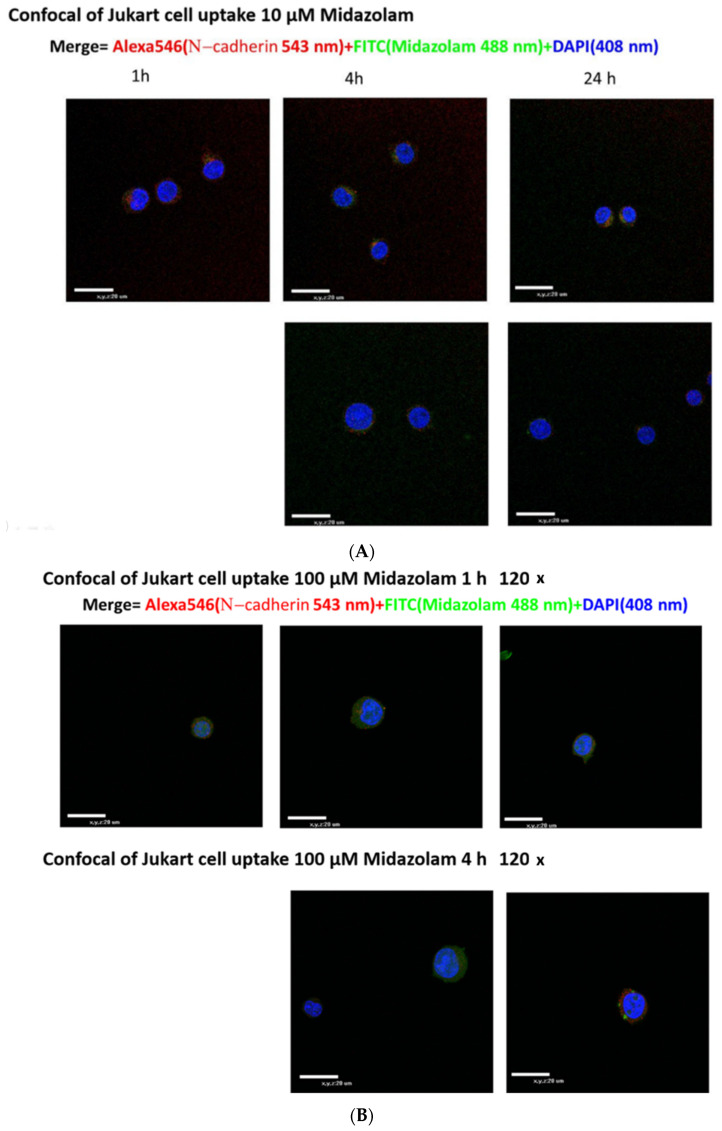
Conjugation of MDZ with Jurkat T-lymphocytes. Scale bar: (x, y, z = 20 μm). (**A**) Confocal fluorescence microscopy picture of Jurkat cells (120×) (1, 4 and 24 h) after treating with FITC-conjugated MDZ (10 μM) stained with Goat anti-rabbit Alexa-546 conjugated IgG. (**B**) Confocal of Jurkat cell (120×) after treating with FITC-conjugated MDZ (100 μM) (1 and 4 h) stained with Goat anti-rabbit Alexa-546 conjugated IgG. The nucleus was stained with DAPI (showing nucleus) to show blue color while Alexa (red showing N-cadherin) and FITC (green showing MDZ) were found on the cell membrane. Co-localization of fluorescence dye was seen starting from 1, 4, and 24 h in both pictures.

Confocal fluorescence microscopy of Jurkat cells (1, 4, and 24 h) after treating with FITC-conjugated MDZ (10 μM) stained with Goat anti-rabbit Alexa-546 conjugated IgG (anti-N-cadherin antibody) showed co-localization of fluorescence dye (Figure 1A). The nucleus was stained with DAPI to show blue color while Alexa (red) and FITC (green) were found on the cell membrane. The same result was found with a higher MDZ (100 μM) dose (Figure 1B). These pictures provided us with evidence that MDZ would conjugate with Jurkat cell membrane in 1, 4, and 24 h.

### 2.2. Effect of MDZ on I_K(DR)_ Recorded from Jurkat T-lymphocytes

**Figure 2:**
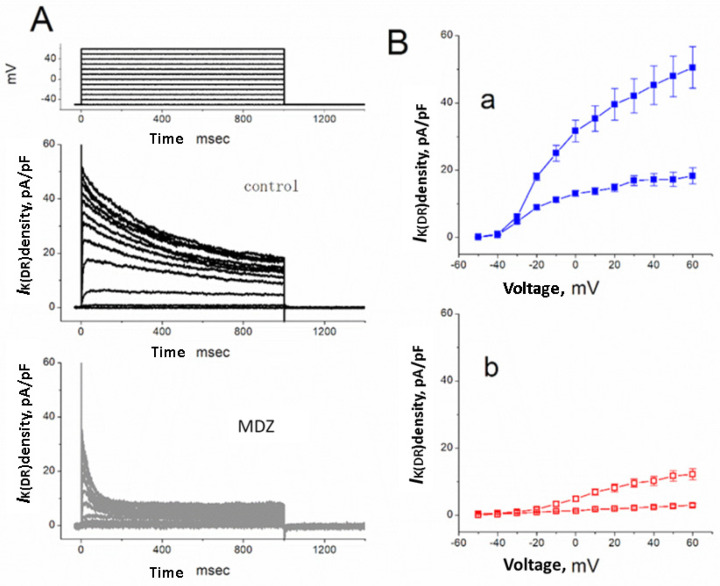
Inhibitory effect of MDZ on *I*_K(DR)_ density in Jurkat T-lymphocytes. In this set of experiments, Jurkat cells were bathed in Ca^+^-free Tyrode’s solution and the recording pipette was filled with K^+^-containing solution described under Materials and Methods. (**A**) Superimposed current densities obtained in the absence (upper) and presence (lower) of 30 µM MDZ. The examined cell was depolarized from −50 mV to a series of the potentials ranging from −50 to +60 mV in 10-mV increments. The uppermost part indicates the voltage protocol examined. (**B**) Average current density versus voltage relations for initial (square symbols) and late (circle symbols) components of *I*_K(DR)_ density in the absence (**Ba**; filled symbols) and presence (**Bb**; open symbols) of 30 µM MDZ (mean ± SEM, *n* = 9–12 for each point).

The whole-cell configuration of the patch-clamp technique was initially utilized to investigate whether MDZ exerted any effects on ion currents in these cells. To record K^+^ outward currents and avoid the contamination of Ca^2+^-activated K^+^ currents, Jurkat cells were bathed in Ca^2+^-free Tyrode’s solution, and the recording pipettes were filled with K^+^-containing solution described in Materials and Methods. As illustrated in Figure 2, when the examined cell was held at −50 mV and depolarizing voltage pulses from −50 to +60 mV in 10-mV increments were applied, a family of outward currents was readily elicited. The threshold for elicitation of these currents was noted to be around −30 mV with a reversal potential of −72 mV. Current magnitude became progressively larger with greater depolarization, indicating that there was an outward rectifying property for these currents. This population of outward currents in these cells was hence referred to as *I*_K(DR)_ and identified to resemble the K_V_1.3-encoded currents [18,24,39]. Moreover, an early study from our laboratory showed that the amplitude of this outward current could be depressed by margatoxin (100 nM), a specific blocker of K_V_1.3-encoded current [18]. Interestingly, as cells were exposed to MDZ (30 µM), the *I*_K(DR)_ density was readily reduced at the voltages ranging between −20 and +60 mV. For example, when the depolarizing pulses from −50 to +50 mV were applied, MDZ (30 µM) significantly decreased *I*_K(DR)_ density measured at the end of voltage pulses by 84.5 ± 4.2% from 517 ± 84 to 80 ± 16 pA (*n* = 12). After wash-out of MDZ, current density at +50 mV returned to 435 ± 41 pA (*n* = 8).

**Figure 3:**
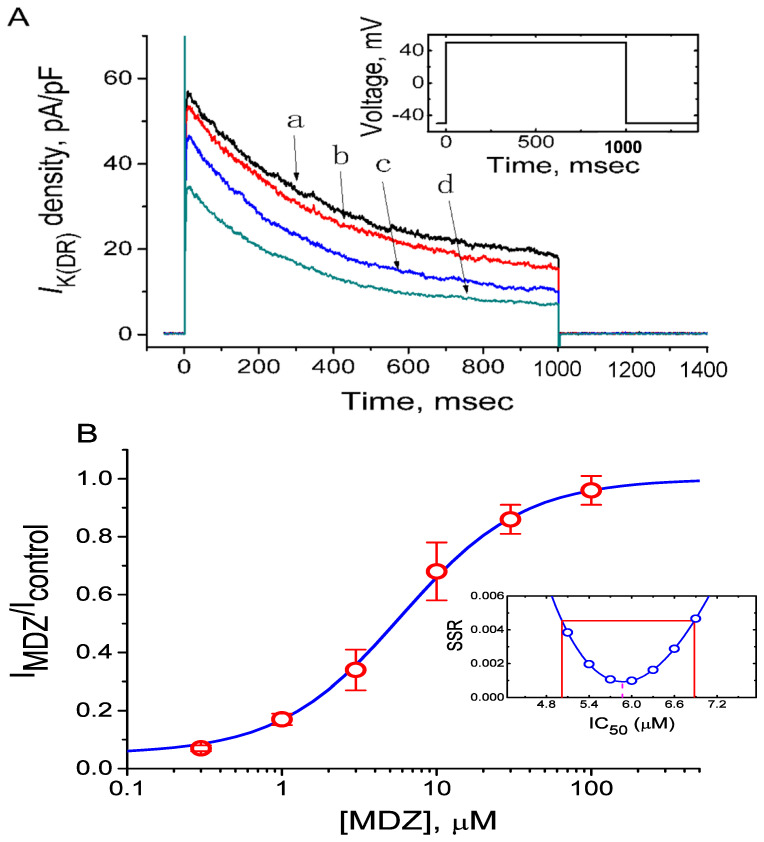
Concentration–response relationship for MDZ-induced inhibition of *I*_K(DR)_ density in Jurkat T-lymphocytes. (**A**) Original current densities of *I*_K(DR)_ obtained with or without addition of MDZ. a: control; b: 3 µM MDZ; c: 10 µM MDZ; d: 30 µM MDZ. Inset in (**A**) indicates the voltage protocol used. (**B**) Concentration–response curve for MDZ-induced inhibition of *I*_K(DR)_ density in Jurkat T-lymphocytes. The *I*_K(DR)_ density measured at the end of depolarizing pulses during cell exposure to MDZ was compared with the control value (mean ± SEM, *n* = 8–13 for each point). The blue smooth line represents a best fit to a Hill function described in Materials and Methods (Equation (1)). The values for IC_50_, maximally inhibited percentage of *I*_K(DR)_ density, and the Hill coefficient were 5.87 µM, 95% (A = 0.95), and 1.1, respectively. Inset in (**B**) shows confidence assessment of best-fit parameter values (i.e., IC_50_). The parameter range corresponds to the approximate 90% confidence intervals. Red line marks the parameter value at which SSR (the sum of squared residuals) amounts to 0.00454.

The relationship between the MDZ concentration and the relative density of *I*_K(DR)_ was constructed and is illustrated in Figure 3. In these experiments, each cell was depolarized from −50 to +50 mV and *I*_K(DR)_ density was measured at the end of the step pulses. As depicted in Figure 3, addition of MDZ to the medium at the concentrations ranging between 0.3 and 100 µM was found to suppress current density in a concentration-dependent manner in Jurkat cells. By the use of nonlinear least-squares fit to the data points (Equation (1)), the IC_50_ value needed to exert its inhibitory effect on *I*_K(DR)_ density was calculated to be 5.87 µM with a Hill coefficient of 1.1, and this agent at a concentration of 100 µM nearly abolished current density. In the SSR plot appearing in inset of Figure 3B, a horizontal line at SSR = 0.00454 was also appropriately made to determine the two IC_50_ values. For a 90% confidence interval, the lower and upper values amounted to 5.02 and 6.88 µM, respectively. Given that a steep slope on both sides of the minimum (as indicated in dashed line), the resultant IC_50_ value was determined with high confidence. Therefore, these findings reflect that this compound has a significant action on the inhibition of *I*_K(DR)_ density in these cells.

### 2.3. Kinetic Analysis of MDZ-Induced Block of I_K(DR)_

**Figure 4:**
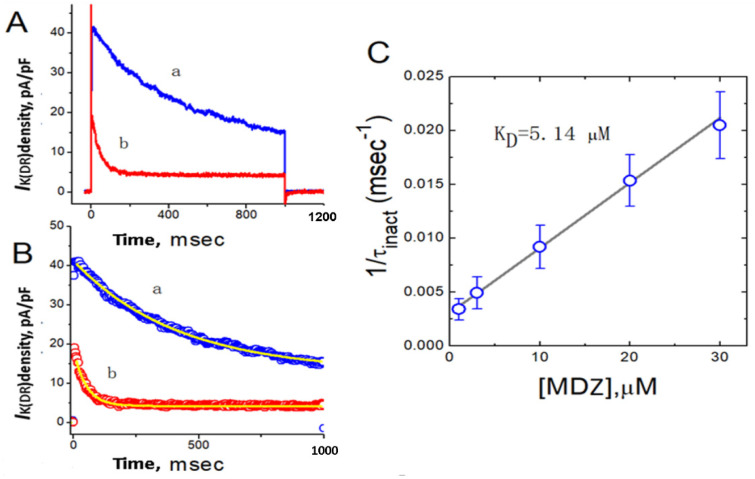
Evaluation of the kinetics of MDZ-induced block of *I*_K(DR)_ density occurring in Jurkat T-lymphocytes. In (**A**), the inactivation time courses of *I*_K(DR)_ density in the absence (a) and presence of 30 µM MDZ (b) were well fitted by a single-exponential shown in (**B**). In these experiments, the cell examined was depolarized from −50 to +50 mV with a duration of 1 sec. In (**C**), the kinetics of MDZ-induced block of *I*_K(DR)_ density in these cells was evaluated. The reciprocal of inactivation time constant of *I*_K(DR)_ density (1/τ_inact_) obtained by exponential fit of the *I*_K(DR)_ trajectory was reliably derived and plotted against the MDZ concentration. The experimental data points were fitted by a linear regression, indicating that MDZ-induced block occurs with a molecularity of 1. Blocking (*k*_+1_) and unblocking (*k*_−1_) rate constants, given by the slope and the *y*-axis intercept of the interpolated line, were 0.000602 msec^−1^M^−1^ and 0.0031 msec^−1^ M^−1^, respectively, and the K_D_ value (*k*_−1_/*k*_+1_ = 5.14 µM) was generated. Each point in (**C**) represents the mean ± SEM (*n* = 11–14).

The biophysical properties of *I*_K(DR)_ density elicited by step depolarization in the presence of the different MDZ concentrations tend to exhibit a pronounced peak followed by an exponential decay as a function of time to a steady-state level. In other words, under our experimental conditions, as a population of K^+^ channels is perturbed by this compound, the current through those channels will change (relax) to a new equilibrium level at a rate which can reflect the underlying kinetics of the channels. For these reasons, we further evaluated the detailed kinetics of MDZ-induced block of *I*_K(DR)_ density in Jurkat T-lymphocytes. The concentration dependence of *I*_K(DR)_-density decay caused by MDZ is illustrated in Figure 4. Although the initial activation phase of *I*_K(DR)_ elicited by depolarizing pulse from −50 to +50 mV remained unchanged during cell exposure to MDZ, its inhibitory effects on *I*_K(DR)_ were noted to be concentration-dependent increase in the rate of current-density decay together with an apparent reduction in the residual, steady-state current. For example, as cells were depolarized from −50 to +50 mV, the inactivation time constants (τ_inact_) of *I*_K(DR)_ in the presence of 10 and 30 µM MDZ were well fitted by a single-exponential with the values of 108.7 ± 18 and 48.8 ± 9 msec (*n* = 11), respectively. Therefore, increasing MDZ concentration not only reduces the peak density of *I*_K(DR)_, but also has the propensity to enhance the apparent inactivation of this current. The results imply that, based on minimal kinetic scheme as described under Materials and Methods (Equation (2)), the inhibitory effect of MDZ on *I*_K(DR)_ density in Jurkat T-lymphocytes can be reasonably explained by a state-dependent block where it has an interaction particularly at the open state of the channel. As predicted by this scheme, the relationship (Equation (3)) between 1/τ_inact_ and (B) (the MDZ concentration) became linear with a correlation coefficient of 0.97 (Figure 4C), and the blocking and unblocking rate constants were calculated to be 0.000602 msec^−1^M^−1^ and 0.0031 msec^−1^, respectively. The *K*_D =_ (*k*_−1_/*k*_+1_) for MDZ-mediated inhibition was computed to be 5.14 µM, a value that is nearly identical with its IC_50_ value (Figure 4). These data thus strengthen the notion that a concentration-dependent increase by MDZ in the rate of *I*_K(DR)_-density inactivation can account for the reduction of *I*_K(DR)_ density in Jurkat T-lymphocytes.

### 2.4. MDZ-Induced Effect on the Steady-State Inactivation Curve of I_K(DR)_ Density

**Figure 5:**
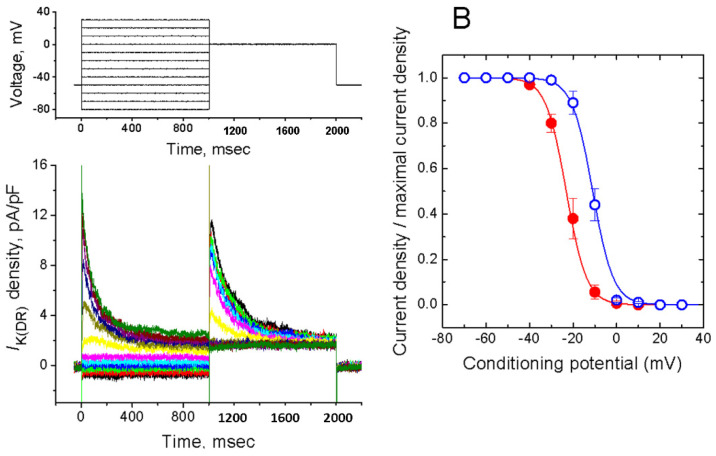
Effect of MDZ on the steady-state inactivation of *I*_K(DR)_ density occurring in Jurkat T-lymphocytes. (**A**) Superimposed *I*_K(DR)_ density obtained in the presence of 30 µM MDZ. The conditioning voltage pulses with a duration of 1 sec to a series of potentials ranging from −80 to +30 mV in 10-mV increments were applied from a holding potential of −50 mV. Following each conditioning pulse, a test pulse to 0 mV with a duration of 1 sec was then employed to elicit *I*_K(DR)_ in these cells. The upper part in (**A**) indicates the voltage protocol used. (**B**) Steady-state inactivation curve of *I*_K(DR)_ density obtained in the absence (O) and presence (●) of 30 µM MDZ (mean ± SEM; *n* = 10–12 for each point).

To characterize inhibitory effect of MDZ on *I*_K(DR)_ density, we also explored the effect of MDZ on the steady-state inactivation of this current density. Figure 5 shows the steady-state inactivation curve of *I*_K(DR)_ density in the absence and presence of MDZ (30 µM). A two-step voltage pulse protocol was applied to the cells. In these experiments, a 1-sec conditioning pulse to different membrane potentials preceded the depolarizing pulse (1 sec in duration) to 0 mV from a holding potential of −50 mV. The interval between two sets of voltage pulses was about 60 sec to prevent incomplete recovery of *I*_K(DR)_ density. The relationships between the conditioning potentials and the normalized density of *I*_K(DR)_ with or without addition of MDZ (30 µM) were plotted and well fitted with the Boltzmann equation as described in materials and methods (Equation (4)). In control, *V*_1/2_ = −11.1 ± 0.6 mV, *k* = 3.86 ± 0.09 mV (*n* = 11), whereas in the presence of 30 µM MDZ, *V*_1/2_ = −22.7 ± 1.1, *k* = 4.17 ± 0.11 (*n* = 10). Therefore, besides its inhibitory action at maximal conductance of *I*_K(DR)_, the presence of MDZ could significantly shift the inactivation curve to a hyperpolarized potential by approximately 12 mV. Conversely, no change in the slope (i.e., *k* value) of the inactivation curve was detected in the presence of this compound.

### 2.5. Inability of Flumazenil to Reverse MDZ-Induced Inhibition of I_K(DR)_

**Figure 6:**
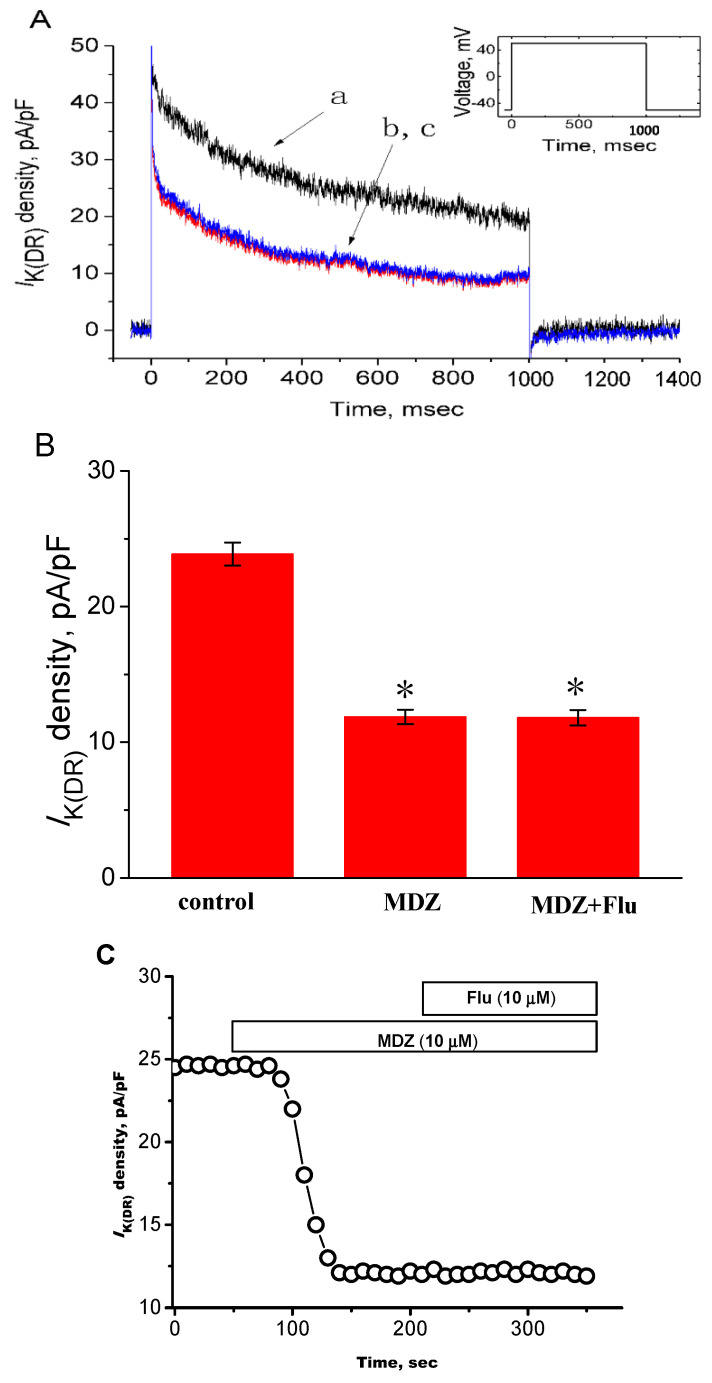
Effect of MDZ and MDZ plus flumazenil on *I*_K(DR)_ density. (**A**) Superimposed current-density traces obtained in the control (a) and during the exposure to 10 µM MDZ (b) and 10 µM MDZ plus 10 µM flumazenil (c). Inset in (**A**) indicates the voltage protocol applied. (**B**) Summary of the data showing effects of MDZ and MDZ plus flumazenil on the *I*_K(DR)_ density measured at the end of depolarizing pulse (mean ± SEM; *n* = 13–14 for each bar). MDZ: 10 µM MDZ; Flu: 10 µM flumazenil. * Significantly different from control (*p* < 0.05). (**C**) Time course in effects of MDZ and MDZ plus flumazenil (Flu) on *I*_K(DR)_ density. Each current density was measured at the end of depolarizing pulse from −50 to +50 mV with a duration of 1 sec. The horizontal bar above indicates the application of 10 μM MDZ or 10 μM flumazenil.

Previous studies have demonstrated that MDZ could suppress functional maturation of murine dendritic cells and perturb the induction by dendritic cells of T helper 1 immunity [7,8]. Those effects appear to be mediated via an interaction of MDZ with peripheral-type benzodiazepine receptors [7,38]. On the other hand, MDZ-induced apoptosis was reported to be unrelated to the binding to central-type benzodiazepine receptors [11]. In this study, we thus explored whether MDZ-induced inhibition of *I*_K(DR)_ density can be influenced by flumazenil, an antagonist of benzodiazepine receptors. However, as shown in Figure 6A, B in continued presence of MDZ, further application of flumazenil (10 µM) to the medium was unable to reverse the inhibition by this compound of *I*_K(DR)_ amplitude. For example, there was no significant difference in *I*_K(DR)_ amplitude between MDZ alone group and MDZ plus flumazenil group (11.9 ± 0.5 pA/pF (in the presence of MDZ), *n* = 14 versus 11.8 ± 0.6 pA/pF (in the presence of MDZ plus flumazenil), *n* = 13, *p* > 0.05). The results led us to suggest that MDZ-induced inhibition of *I*_K(DR)_ in these cells is unlinked to its propensity to interact with benzodiazepine receptors. Figure 6C depicts the time course effects of MDZ and MDZ plus flumazenil on *I*_K(DR)_ density. Additionally, in cells exposed to flumazenil (10 μM) for 1 h, the application of MDZ (10 μM) was still effective at inhibiting *I*_K(DR)_ density (11.9 ± 0.5 pA/pF (in control cells) versus 11.8 ± 0.6 pA/pF (in flumazenil-treated cells), *n* = 8, *p* > 0.05).

### 2.6. Inhibitory Effect of MDZ on IK_Ca_ Channels Recorded from JURKAT T-Lymphocytes

**Figure 7:**
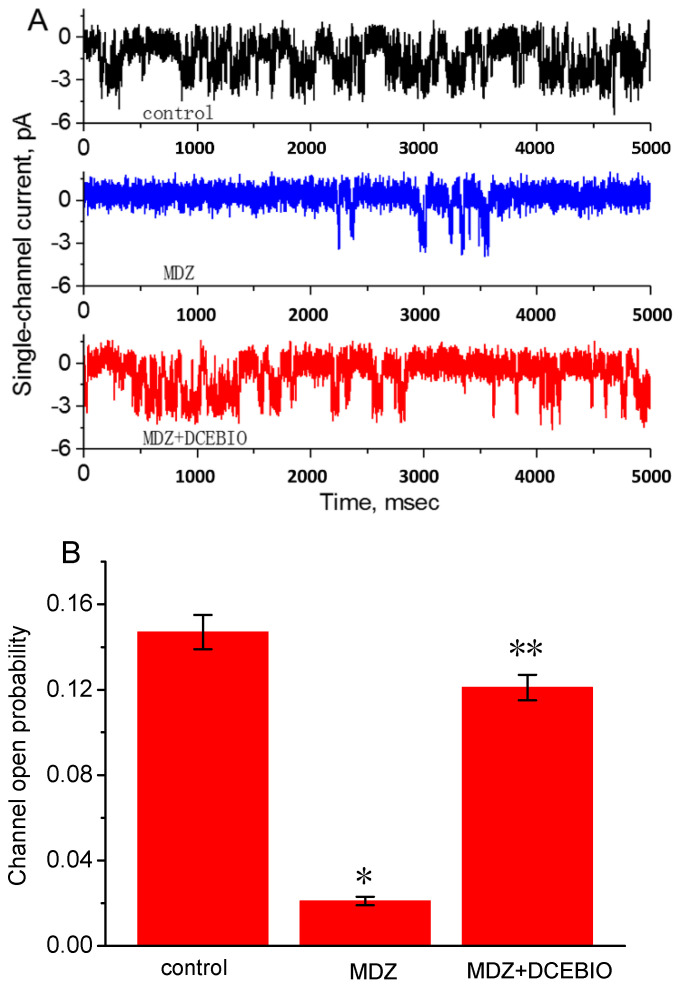
Effect of MDZ on the activity of IK_Ca_ channels in Jurkat T-lymphocytes. In these experiments, cells were bathed in high-K^+^ (145 mM) solution containing 1.8 mM CaCl_2_. Cell-attached current recordings were made, and each cell was constantly held at −60 mV. (**A**) Original IK_Ca_-channel currents obtained in control, during exposure to MDZ (30 µM) and MDZ plus DCEBIO (3 µM). Download deflection indicates the opening event of the channel. (**B**) Summary of the data showing the effects of MDZ and MDZ plus DCEBIO on the probability of IK_Ca_-channel openings (mean ± SEM; *n* = 10–12 for each bar). MDZ: 30 µM MDZ; DCEBIO: 3 µM DCEBIO. * Significantly different from control (*p* < 0.05). ** Significantly different from MDZ (30 µM) alone group (*p* < 0.05).

Previous studies have shown the appearance of IK_Ca_-channel activity in immune cells including lymphocytes [20,27,28,32,33]. The IK_Ca_ channels are encoded by the *KCNN4* gene. There is growing evidence showing that the activity of these channels is intimately linked to lymphocyte reactions [20,27,32,34,40]. We therefore evaluated whether MDZ has any effects on the activity of these channels in Jurkat cells. In these experiments, lymphocytes were bathed in high-K^+^ solution containing 1.8 mM CaCl_2_ and the cell-attached current recordings were performed in these cells. As shown in Figure 7, under symmetrical K^+^ (145 mM) conditions, the activity of IK_Ca_ channels could be readily detected when the cell examined was held at −60 mV. Addition of MDZ was noted to suppress channel activity significantly, while no modification in single-channel conductance of IK_Ca_ channels was demonstrated in the presence of this compound. For example, MDZ at a concentration of 30 μM progressively decreased the channel open probability by 85.7 ± 2.5 % from 0.147 ± 0.008 to 0.021 ± 0.002 (*n* = 12). Moreover, in continued presence of 30 μM MDZ, further addition of DCEBIO, an activator of IK_Ca_ channels [29,31,41], was capable of reversing MDZ-induced reduction of IK_Ca_-channel activity, as evidenced by a significant elevation of channel open probability to 0.121 ± 0.006 (*n* = 10). Similar to the effect of MDZ, the addition of TRAM-34 (3 μM), a blocker of IK_Ca_ channels [30,31], was effective at decreasing the probability of IK_Ca_-channel openings (data not shown). However, the single-channel conductance of IK_Ca_ channels obtained between the absence and presence of MDZ (30 μM) did not differ significantly (34.4 ± 0.8 pS (in the absence of MDZ), *n* = 10 versus 33.1 ± 0.9 pS (in the presence of MDZ), *n* = 9, *p* > 0.05). Therefore, it is clear from these results, like the *I*_K(DR)_ amplitude, the activity of IK_Ca_ channels in Jurkat T-lymphocytes can be suppressed by MDZ.

### 2.7. Effect of MDZ on I_K(DR)_ in PHA-Preactivated Human T Lymphocytes

**Figure 8:**
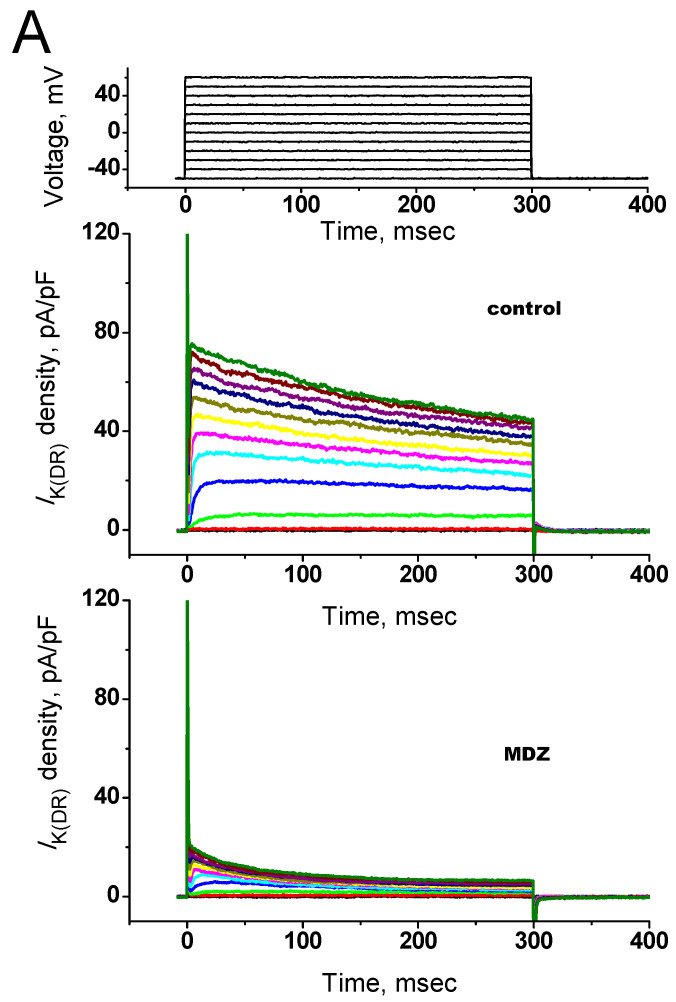

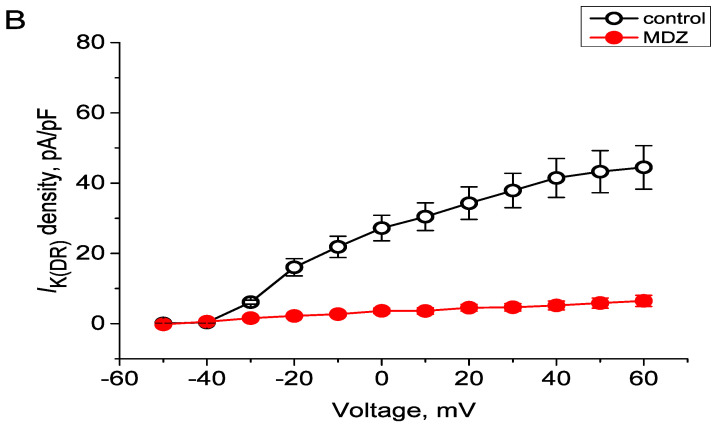
Effect of MDZ on *I*_K(DR)_ density recorded from PHA-preactivated human T lymphocytes. (**A**) Superimposed current traces obtained in the absence (upper) and presence (lower) of 30 µM MDZ. The uppermost part indicates the voltage protocol used. (**B**) Average current density versus voltage relationships of *I*_K(DR)_ measured at the end of depolarizing pulses (mean ± SEM; *n* = 7–9 for each point). O: control; ●: in the presence of 30 µM MDZ.

Next, we explored whether MDZ exerted any effects on human T lymphocytes, because Jurkat T-lymphocytes are individual cells of the human T-leukemia-derived line [15]. As illustrated in Figure 8, MDZ (30 μM) was found to suppress the density of *I*_K(DR)_ occurring in PHA-preactivated T lymphocytes significantly, which was accompanied by the decreased time constant of current-density decay in response to membrane depolarization. For example, as cells were exposed to MDZ (30 μM), *I*_K(DR)_ elicited by membrane depolarization from −50 to +50 mV decreased from 43.3 ± 6.1 to 5.8 ± 1.5 pA/pF (*n* = 7, *p* < 0.05). Concomitant with these results, the time constant of *I*_K(DR)_ activation in response to membrane depolarization was decreased to 78 ± 9 msec (*n* = 7, *p* < 0.05) from a control value of 158 ± 11 msec (*n* = 8). Therefore, similar to the results described in Jurkat T-lymphocytes, MDZ was thus effective at suppressing *I*_K(DR)_ density in PHA-preactivated human T lymphocytes.

### 2.8. Effect of LPS Challenge on the Levels of IL-6 in JUKART Cell

**Figure 9:**
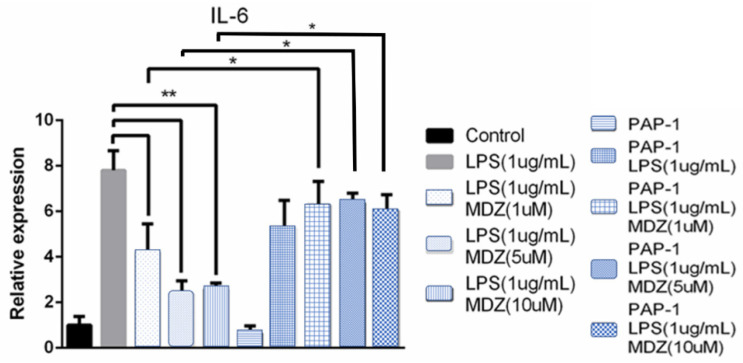
Effect of PAP-1(Kv1.3 channel blocker) on cytokine (IL-6) expression on MDZ (1, 5 and 10 µM) treated Jurkat T-lymphocytes. *n* = 3, data were mean +/- SEM, PAP-1 + LPS + MDZ (1, 5, 10 µM) vs. LPS + MDZ (1, 5, 10 µM) group, * *p* < 0.05; LPS vs. LPS + MDZ(1, 5, 10 µM) group, ** *p* < 0.05 (ANOVA followed by Tukey’s post hoc test).

In a final set of experiments, we examined whether MDZ could affect immune-regulating cytokine expression in LPS-treated human T lymphocytes by using Semi-quantitative RT-PCR. As demonstrated in Figure 9, the results revealed that LPS treatment enhanced IL-6 expression, but such enhancement was suppressed significantly by MDZ (1, 5 and10 µM) in Jurkat T-lymphocytes. Our current data support the idea that MDZ could regulate immunological responses of lymphocytes and may further affect lymphocyte immune functions.

Next, we checked if pretreatment with PAP-1 (Kv1.3 channel blocker) could rescue the effect of MDZ on cytokine expression in LPS-treated human T-lymphocytes. Results showed that pretreatment with LPS + PAP-1 + MDZ(1 μM) restored IL-6 expression (*p* < 0.05) and the rescue effect was consistent with increasing MDZ (5 and 10 µM) concentration (Figure 9).

## 3. Discussion

Our study indicates that MDZ, a benzodiazepine sedative, is capable of inducing the blockade of *I*_K(DR)_ currents and elevating the rate of *I*_K(DR)_ inactivation in Jurkat T-lymphocytes. This compound suppressed *I*_K(DR)_ density in these cells in a concentration-, time- and state-dependent fashions. Inability of flumazenil to reverse MDZ or diazepam-induced inhibition of *I*_K(DR)_ density was observed in this study. MDZ or diazepam also decreased the activity of IK_Ca_ channels significantly. Therefore, these agents have an important profile in that they suppress *I*_K(DR)_ density and the activity of IK_Ca_ channels via the mechanisms other than an interaction at peripheral benzodiazepine receptors. It is reasonable to assume that their effects on the activity of K^+^ channels could possibly result from an interference with a channel-associated binding site. In addition to its effects on the signaling of GABA_A_ receptor pathway [38], MDZ-induced blockade of these ion channels could be another intriguing mechanism through which it influences the functions of lymphocytes, if similar findings occur in vivo. Given that the importance of *I*_K(DR)_ (i.e., K_V_1.3-encoded current) in contributing to functional activities of lymphocytes [15,18,20,22,24,36], it is also conceivable that MDZ-induced block of *I*_K(DR)_ is involved in any alteration in immunological reactions, such as platelet-leukocyte aggregation and cell apoptosis [4,9].

In parallel with previous observations [17,18,36,42], the biophysical properties of *I*_K(DR)_ observed in Jurkat T-lymphocytes or PHA-preactivated human T lymphocytes closely resemble those of K_V_1.3-encoded current or via a heterologous expression system including K_v_1.3/K_v_1.5 hybrid channels [43]. Under our experimental conditions, the addition of MDZ inhibited *I*_K(DR)_ density in a concentration-dependent manner with an IC_50_ value of 5.87 μM. This value was noted to agree with the *K*_D_ value (5.14 μM) which was calculated on the basis of the first-order blocking scheme described in Materials and Methods (Equations (2) and (3)). Therefore, the results indicate that MDZ could act as a state-dependent blocker of *I*_K(DR)_ density existing in Jurkat T-lymphocytes. In particular, this value is similar to those required for its induction of apoptotic changes or of alterations in dendritic cells [4,7]. The observed effects of MDZ in this study should thus occur at a concentration achievable in humans.

A hyperpolarized shift in the steady-state inactivation curve of *I*_K(DR)_ density in the presence of MDZ was actually observed in Jurkat T-lymphocytes, despite its failure to alter the slope factor of this curve. In addition, we also found the ability of this compound to cause the time-dependent block of *I*_K(DR)_ density. Unlike MDZ, dexmedetomidine, another intravenous sedative with the agonistic activity of α_2_-adrenergic receptors, did not produce any changes in the inactivation time course of *I*_K(DR)_ density in Jurkat T-lymphocytes, although it suppressed *I*_K(DR)_ density (data not shown). The rate of current-density inactivation was considerably enhanced as the MDZ concentration increased. Assuming that periodical changes in membrane potential of inner mitochondrial membranes may take place [44], the issue of whether blockade of mitochondrial K_V_1.3 channels caused by MDZ is responsible for its effects on Bax-K_V_1.3 interactions [23,37] is worthy of being investigated. Nonetheless, because of the importance of *I*_K(DR)_ (i.e., K_V_1.3-encoded current) in contributing to functional activities of lymphocytes [19,36], the effects presented herein could provide novel insights into pharmacological or immunological properties of MDZ and other structurally related compounds (e.g., diazepam) [1,2,4,5,6,9,20,45].

Inhibition of K^+^ channels (i.e., K_V_1.3 and IK_Ca_ channels) caused by MDZ might be intimately linked to the proapoptotic activities of this agent. Recent studies have demonstrated that K_V_1.3 channels are functionally expressed not only in the plasmalemma but also in the inner mitochondrial membrane of lymphocytes [19,37]. Inhibition of mitochondrial K_V_1.3 channels inherent in T lymphocytes by a proapoptotic protein Bax appears to be the first crucial event in the process of an activation of the mitochondrial pathway connected with apoptosis [37]. Consequently, it is conceivable that a blockade of mitochondrial K_V_1.3 channels in Jurkat T-lymphocytes may mimic the effects of Bax and trigger the apoptotic cell death through an activation of the mitochondrial pathway [23,36,39]. 

A previous report by Nakamura et al. (2007) showed that MDZ could suppress vascular ATP-sensitive K^+^ (K_ATP_) channels. In our study, the internal solution used for whole-cell current recordings contained ATP at a concentration of 3 mM. Neither diazoxide (30 μM) nor pinacidil (30 μM), which are activators of K_ATP_ channels, reversed MDZ-induced inhibition of *I*_K(DR)_ (data not shown). Therefore, the observed effect of MDZ on inhibition of *I*_K(DR)_ in Jurkat T-lymphocytes is unlikely to be mediated through its blockade of either K_ATP_ channels or large-conductance Ca^2+^-activated K^+^ channels [12,13].

With the aid of Blastx program (http://blast.ncbi.nlm.nih.gov/ Accessed date: 31 August 2016), we examined the similarity of amino acid sequence between peripheral-type benzodiazepine receptor (AAA03652.1) and K_V_1.3-encoded protein (KCNA3; NP_002223.3). Interestingly, a portion of peripheral-type benzodiazepine receptor, to which the sequence of KCNA3 shares the similarity (63%), is located at 3–10, while that of KCNA3 protein is at 90–97. It is thus anticipated that the molecules of MDZ or structurally similar compounds may interact at these regions to influence the kinetics of *I*_K(DR)_ or/and peripheral-type benzodiazepine receptor.

The K_V_1.3 channels have been demonstrated to be expressed in inner mitochondrial membrane of many types of neoplastic cells [19,39]. It is tempting to speculate that MDZ and other structurally related compounds can diffuse across the plasma membrane and reach intracellular compartments, thereby inhibiting mitochondrial K_V_1.3 channels and perturbing the Bax-K_V_1.3 interaction [39]. Whether the ability of this compound to induce apoptosis described previously in mouse Leydig cells [44] is connected with its inhibition of plasmalemmal or mitochondrial K channels has yet to be clearly demonstrated.

Suppression of IL-6 production by MDZ in different cell types including glioma cell, Jurkat-T cell or human peripheral blood mononuclear cells (PBMCs) had been previously reported [46,47,48]. Our results for IL-6 by semi-quantitative RT-PCR matched their findings with LPS activated macrophages. Study by Takaono [49] showed that MDZ (1.5 mg/mL) interfered with the production of IL-6 by LPS-stimulated PBMCs from healthy volunteers. Continuous infusion (48 h) of MDZ in critically ill surgical patients also showed suppression of the pro-inflammatory cytokines IL-1b, IL-6 and TNF-α and IL-8 production [50]. Result by Miyawaki showed that suppression of cytokines secretion by MDZ was not via either central-type or peripheral type benzodiazepine receptors [45]. Kazama in his review article also stated the presence of K_V_1.3 channels in T lymphocytes [51]. Results from our experiment showed that MDZ could conjugate with Jurkat T-lymphocytes and suppress the production of IL-6. LPS + PAP-1 pretreatment resulted in rescuing IL-6 inhibition by MDZ provided evidence that MDZ suppression of IL-6 production might be mediated through Kv1.3 channel.

The exposure to MDZ was able to decrease the probability of IK_Ca_-channel openings effectively in Jurkat cells; however, it caused no changes in single-channel conductance of these channels. A current study showed that the ability of MDZ to suppress the apoptosis of astrocytes induced by oxygen glucose deprivation, which could be mediated through a mechanism linked to JAK2-STAT3 signaling pathway [10]. The activity of IK_Ca_ channels was also reported to be functionally expressed in astrocytes [29,35]. To what extent the IK_Ca_-channel activity in astrocytes contributes to MDZ mediated regulation of apoptotic changes thus remains to be further determined. Taken together, despite the detail mechanism of MDZ actions on these K^+^ channels being unclear, if similar findings are observed in lymphocytes occurring in cell culture or in vivo to those described herein, the actions of MDZ or its functionally related agents (e.g., diazepam) observed in the present study will result in significant changes in immune reactions [2,4,5,6,23].

## 4. Materials and Methods

### 4.1. Drugs and Solutions

#### Midazolam

(MDZ; 8-chloro-6-(2-fluorophenyl)-1-methyl-4H-imidazo [1,5-a][1,4] benzodiazepine; Dormicum^®^; Midatin^®^) was obtained from Nang Kuang Pharmaceutical Co. (Tainan City, Taiwan). Dexmedetomidine was obtained from Abbott Laboratories (Abbott Park, IL), diazepam, diazoxide, flumazenil, phytohemagglutinin (PHA) and pinacidil were from Sigma-Aldrich (St. Louis, MO), DCEBIO (5,6-dichloro-1-ethyl-1,3-dihydro-2*H*-benzimidazol-2-one) and TRAM-34 (1-((2-chlorophenyl) (diphenyl)methyl)-1*H*-pyrazole) were from Tocris Cookson Ltd. (Bristol, UK), and margatoxin was from Alomone Labs (Jerusalem, Israel). For cell preparations, culture media, fetal bovine serum (FBS), L-glutamine, trypsin/EDTA, penicillin-streptomycin, and amphotericin B were obtained from Invitrogen (Carlsbad, CA, USA). All other chemicals were obtained from regular commercial chemicals and of reagent grade. Reagent water was obtained using a Milli-Q ultrapure water purification system (Millipore, Bedford, MA, USA).

The composition of bathing solution (i.e., normal Tyrode’s solution) was as follows: 136.5 mM NaCl, 5.4 mM KCl, 1.8 mM CaCl_2_, 0.53 mM MgCl_2_, 5.5 mM glucose, and 5.5 mM HEPES-NaOH buffer, pH 7.4. To measure K^+^ currents, the patch pipette was backfilled with a solution consisting of 130 mM K-aspartate, 20 mM KCl, I mM KH_2_PO_4_, 1 mM MgCl_2_, 3 mM Na_2_ATP, 0.1 mM Na_2_GTP, 0.1 mM EGTA, and 5 mM HEPES-KOH buffer, pH 7.2. To avoid the contamination of Cl^-^ currents, Cl^-^ ions inside the pipette solution were replaced with aspartate. For the recordings of IK_Ca_-channel activity in Jurkat cells, high K^+^-bathing solution was used, and its composition was 145 mM KCl, 0.53 mM MgCl_2_, and 5 mM HEPES-KOH buffer, pH 7.2, while the pipette solution contained 145 mM KCl, 2 mM MgCl_2_, and 5 mM HEPES-KOH buffer, pH 7.2. The pipette solution was filtered on the day of use with a syringe filter of 0.22 μm pore size (Millipore).

### 4.2. Cell Preparations

The Jurkat T cell line, a human T cell lymphoblast-like cell line (clone E6-1), was obtained from the Bioresource Collection and Research Center (BCRC-60255; Hsinchu, Taiwan). Cells were routinely grown in RPMI-1640 medium supplemented with 10% heat-inactivated FBS (*v/v*), 100 U/mL penicillin and 10 μg/mL streptomycin. They were maintained at 37 °C in a 95% air and 5% CO_2_ humidified atmosphere. Cell viability was often evaluated by the trypan blue-exclusion test. The experiments were made five or six days after cells had been cultured (60%–80% confluence). For preparations of human T lymphocytes, CD3^+^ and CD4^+^ lymphocytes were isolated from healthy donors by E-rosetting (StemCell Technology, Vancouver, Canada) and Ficoll-Plaque density-gradient centrifugation (ICN Biomedicals, Aurora, OH, USA). Freshly isolated human T lymphocytes were preactivated with 4 μg/mL PHA for about four hours. Blood intended for the isolation of T lymphocytes was obtained from healthy volunteers.

### 4.3. Electrophysiological Measurements

Before the experiments were performed, cells were harvested with 1% trypsin/EDTA solution and aliquot of cell suspension was subsequently transferred to a home-made recording chamber positioned on the stage of a DM-IL inverted microscope (Leica Microsystems, Wetzlar, Germany) coupled to digital video system (DCR-TRV30; Tokyo, Japan) with a magnification of up to 1500×. Cells were immersed at room temperature (20–25 °C) in normal Tyrode’s solution containing 1.8 mM CaCl_2_. The recording pipettes were pulled from Kimax-51 glass capillaries (#34500; Kimble Glass, Vineland, NJ, USA) using either a two-step microelectrode puller (PP-83 or PP-830; Narishige, Tokyo, Japan) or a P-97 Flaming/Brown micropipette puller (Sutter, Novato, CA, USA), and their tips were fire-polished with a microforge (MF-83; Narishige, Tokyo, Japan). As the pipettes were filled with different internal solutions described above, their resistance commonly ranged between 3 and 5 MΩ. During each experiment, the pipette used was mounted on and manipulated by a WR-98 hydraulic micromanipulator (Narishige, Tokyo, Japan). Current signals recorded under cell-attached or whole-cell configuration were measured with standard patch-clamp technique by use of either an RK-400 amplifier (Bio-Logic, Claix, France) or an Axopatch-200B amplifier (Molecular Devices, Sunnyvale, CA, USA) [28,30]. Liquid junction potential existing between the internal pipette solution and extracellular medium were corrected immediately before seal formation was made.

### 4.4. Data Recordings

The data were stored online in an ASUSPRO-BU401LG computer (ASUS, Taipei City, Taiwan) at the sampling rate of 10 kHz. The computer was equipped with a Digidata-1440A interface device (Molecular Device), through which analog-to-digital and digital-to-analog conversions were controlled by pCLAMP 10.2 software (Molecular Devices, Sunnyvale, CA, USA). Current signals were low-pass filtered at 3 kHz. The signals achieved during the experiments were analyzed off-line by use of various analytical tools including LabChart^TM^ 7.0 program (AD Instruments; Gerin, Tainan City, Taiwan), Origin^®^ 8.0 (OriginLab, Northampton, MA, USA) and custom-made macro procedures built in Excel 2013 (Redmond, WA, USA). Different voltage profiles utilized in this study (e.g., one- or two-pulse experiments) were appropriately created from pCLAMP 10.2 and, through digital-to-analog conversion, they were applied to evaluate the current-voltage (*I-V*) relationship, the steady-state inactivation curve, or the recovery of current inactivation for ion currents (e.g., *I*_K(DR)_).

### 4.5. Conjugation of MDZ with FIuorescein Dye for Confocal Microscopy Imaging

Fluorescein isothiocyanate (FITC) was obtained from Sigma-Aldrich (St. Louis, MO, USA). 1-ethyl-3-(3-dimethylaminopropyl) carbodiimide (EDC) was from Sigma-Aldrich (St. Louis, MO, USA). The entire conjugation process was performed for about 12 h. Briefly, MDZ (pH2.0) 5 mg were dissolved in 1 mL PBS solution before use. Excess 1-ethyl-3-(3-dimethylaminopropyl)carbodiimide hydrochloride (EDC) was added to 5.45 mg FITC (ratio of 20:1) in 1 ml MQ water. The solutions were stirred at room temperature for 10 min before MDZ (pH2.0) solution was added. The mixture was then stirred for 2 h in the dark. The final solution of MDZ–FITC was regulated to pH 7.0. Jurkat cells (1 × 10^6^) are cultured in a 6-well dish in 3 mL RPMI medium (Gibco, ThermoFisher, Waltham, MA, USA) with10% fetal bovine Serum (FBS) (Gibco, ThermoFisher, Waltham, MA., USA). After treating with FITC-conjugated MDZ (final conc. = 10 µM) for 1, 4, 24 h, cells are centrifuged under 1200 rpm and collected. Cells were washed once in phosphate-buffered saline (PBS) and fixed in 4% paraformaldehyde for 40 min at room temperature. After washed by PBS 3 × 5 min, cells were blocked with 0.5% BSA (Sigma-Aldrich, St. Louis, MO, USA) with 0.5% Tween-20 (Sigma-Aldrich, St. Louis, MO, USA) and 0.1%Triton X-100 (Sigma-Aldrich, St. Louis, MO, USA) in PBS (PBSTT) for 1 h. Then cells were washed by PBST (0.5% Tween-20 in PBS) three times and incubated with anti-N-cadherin antibody (Anti-N Cadherin antibody (EPR1791-4) (ab76011), EPIC MIC, Abcam, UK) in PBS-T (1:100) at 4 °C overnight. After washing with PBST for three times, the cells were stained with Goat anti-rabbit Alexa-546 conjugated IgG (1:250) (Invitrogen, Carlsbad, CA, USA) for 1 h at room temperature. Cells were washed with PBS three times and VECTA SHIE LD antifade mounting medium with 4′6-diamidino-2-phenylindole (DAPI, Vector Laboratories Inc., Burlingame, CA, USA) to show the nuclei. The cover slides were mounted with nail polish. The localization of MDZ and N-cadherin are detected directly under a microscopy with a Confocal laser microscope system C1si (Nikon C1-Si confocal microscope, Tokyo, Japan).

### 4.6. RNA Extraction and Reverse Transcriptase-Polymerase Chain Reaction (RT-PCR)

Total RNA from cultured cells was extracted by TRIzol reagent (Invitrogen, Carlsbad, CA, USA) and the mRNA (2 μg) was synthesized to cDNA using reverse transcriptase kits (Clontech, Mountain View, USA) according to the manufacturer’s protocol. Briefly, RNA samples were heated with RNase-free H2O and random primers at 70 °C for 3 min and immediately cool on ice. A mixture containing dNTPs, 5× first-strand buffer, DTT, and MMLV reverse transcriptase was subsequently added by gently mix and then incubated at 42 °C for 60 min followed with terminating the reaction by heating at 70 °C for 15 min. RT-PCR was performed on the Thermo Cycle (Applied Biosystems, Bedford, MA, USA) using 2x Taq DNA Polymerase Mastermix (Bioman, Guangzhou, China). The PCR conditions were 95 °C for 1 min followed by 95 °C for 1 min, 55 °C for 2 min and 70 °C for 1 min for 30 cycles; and 70 °C for 2 min, followed by a immerse at 4 °C until the products were run on a 1% agarose gel. The sequences of primers used in this experiment are summarized as follows (Table 1):

The housekeeping gene, β-actin, was used to normalize all test genes. Data were analyzed using ImageJ software (version 1.41) from W. Rasband (National Institutes of Health, Bethesda, MD) (http://rsbweb.nih.gov/ij/, accessed date: 31 August 2016) and results are expressed as fold difference.

Jurkat T cells (1 × 10^5^) were seeded in 6 wells (medium 2 mL) while 5 mg PAP-1(Catalog No. A11521, Adooq Bioscience, Irvine, CA, USA) was dissolved in DMSO (2.85 mL) and prepared into 5 mM stocking solution. Concentration of PAP-1 was 20 µM (concentration of DMSO <0.05%). Concentration for LPS 1 ug/mL (stock: 5 mg/mL). Six hours after seeding, cells were pretreated with LPS (2 or 4 h) and PAP-1 was subsequently added to the wells for 30 min. Cells were harvested for RNA extraction after exposure to MDZ (1, 5 and 10 μM) for 24 h.

### 4.7. Data Analyses

To determine concentration-dependent inhibition of MDZ on the amplitude of *I*_K(DR)_, cells were bathed in Ca^2+^-free Tyrode’s solution and the depolarizing pulses from −50 to +50 mV with a duration of 1 sec at a rate of 0.05 Hz were applied. The *I*_K(DR)_ amplitude measured at the end of +50 mV during cell exposure to different concentrations (0.3–100 μM) of this compound was thereafter compared with the control value. To ensure accurate fitting, the concentration-dependent relation of MDZ on inhibition of *I*_K(DR)_ was fit using a modified form of sigmoidal Hill equation:

(1) $$\frac{I_{\mathrm{MDZ}}\mathrm{density}}{I_{\mathrm{control}}}=A\times \left(\frac{{\left[\mathrm{MDZ}\right]}^{n_{H}}}{{\mathrm{IC}}_{50}^{n_{H}}+{\left[\mathrm{MDZ}\right]}^{n_{H}}}\right)+a$$

where (MDZ) indicates the MDZ concentration; IC_50_ and n_H_ are the concentration needed for a 50% inhibition and Hill coefficient, respectively; *A* and *a* denote the maximal fraction of total current density, which is MDZ sensitive and insensitive, respectively.

According to a minimal kinetic scheme described previously [18], inhibitory action of MDZ on *I*_K(DR)_ density detected in Jurkat T-lymphocytes can be explained primarily by a state-dependent blocker that binds to the open state of the channel. The model can be drawn in the form of state diagram and described by

(2) $$C\;\overset{\alpha }{\underset{\beta }{⇄}}\;O\;\overset{k_{+1}\cdot [B]}{\underset{k_{-1}}{⇄}}\;O\cdot B$$

where α and β indicate the voltage-dependent rate constants for the opening and closing of K^+^ channels, *k*_+1_ and *k*_−1_ are those for blocking and unblocking by MDZ, and [B] is the MDZ concentration. C, O and O·B shown in diagram denote the closed, open, and open-blocked states of the channel, respectively. The blocking and unblocking rate constants, *k*_+1_ and *k*_−1_, were determined from the inactivation time constants (*τ_inact_*) of *I*_K(DR)_ in response to depolarizing pulses during cell exposure to different concentrations of MDZ. Blocking and unblocking rate constants were evaluated using the relation:

(3) $$\frac{1}{\tau_{inact}}=k_{+1}\times [B]+k_{-1}$$

where *k*_−1_ and *k*_+1_, respectively, indicate the intercept with the vertical axis at [*B*] = 0 and the slope (steepness of a line) of the linear regression which interpolates the reciprocal time constant of *I*_K(DR)_-density inactivation (1/τ_inact_) versus different MDZ concentrations (1–30 μM). Such a linear relationship can be used to estimate an average rate of change. The *k*_−1_ divided by *k*_+1_ can result in the value of dissociation constant (*K*_D_) [18,51].

The steady-state inactivation curve of *I*_K(DR)_ density with or without addition of MDZ was plotted against the test potential and fit to the Boltzmann equation:

(4) $$\frac{I\;\mathrm{density}}{{I\;\mathrm{density}}_{\max}}=\frac{1}{1+e^{\left(\frac{V-V_{1/2}}{k}\right)}}$$

where *V* is the conditioning potential in mV, *V*_1/2_ is the membrane potential for half-maximal inactivation, and *k* is the slope factor (i.e., the steepness) for the inactivation curve of *I*_K(DR)_ density. The Solver subroutine built under Microsoft Excel was implemented to fit the data points by least-squares minimization procedure as described previously [52].

### 4.8. Single-Channel Analyses

Single IK_Ca_-channel amplitudes recorded from Jurkat cells were commonly analyzed using pCLAMP 10.2 (Molecular Devices, Sunnyvale, CA, USA). Multigaussian adjustments of the amplitude distributions among channels were appropriately created to determine unitary currents. Functional independence among channels was validated by comparing the observed stationary probabilities with the values calculated according to binominal law. The probabilities of IK_Ca_-channel openings were reliably estimated using an iterative process to minimize the χ^2^ values calculated with a sufficient large number of independent events. By use of linear regression, the single-channel conductance of IK_Ca_ channels with or without addition of MDZ was calculated using mean unitary amplitudes measured at different levels of voltage [28,30].

### 4.9. Statistical Analyses

The averaged results are presented as the mean ± standard error of the mean (SEM) with sample sizes (*n*) indicating the cell number from which the data were taken, and the error bars appearing in each figure are plotted as SEM. The paired or unpaired Student’s *t*-tests were used for statistical analyses. As the statistical difference among different groups was necessarily evaluated, post-hoc Duncan’s multiple comparisons were further performed. To assess the sum of squared residuals (SSR) as a function of IC_50_ value for inhibitory action of MDZ on *I*_K(DR)_ density, the 90% confidence interval was appropriately estimated using Fisher’s *F* distribution (i.e., FINV function embedded in Microsoft Excel). The confidence assessment of best-fit parameter values (e.g., IC_50_) was then reliably evaluated [18,30]. Statistical analyses were made using IBM SPSS version 17 (Armonk, New York, NY, USA). Statistical significance was determined at a *p* value of <0.05.

## 5. Conclusions

MDZ, a common sedative drug used during anesthesia or intensive care, could significantly suppress current density of *I*_K(DR)_ in concentration-, time-, and state-dependent manners in Jurkat T-lymphocytes and depress immune-regulating cytokine (IL-6) expressions in LPS-treated human T lymphocytes in this study. This study provides evidence for the possible mechanism of MDZ immune suppression (IL-6) through Kv1.3 channel. Although the mechanism or ion channels leading to this immune suppression both in vitro and in vivo still require further studies, physicians should pay more attention to critical or immune suppressive patients when prescribing MDZ to them.

## Figures and Tables

**Table 1:** The sequences of primers used in this experiment are summarized as follows.

Primer Sequence (5′–3′)		
	Forward	Reverse
hIL-6 (360 bp)	TTC GGT CCA GTT GCC TCT C	TGG CAT TTG TGG TTG GGT CA
hIL-8 (300 bp)	AAG AGA GCT CTG TCT GGA CC	GAT ATT CTC TTG GCC CTT GG
β-actin (520 bp)	GCTGGAAGGTGGACAGCGAG	TGGCATCGTGATGGACTCCG

## Abbreviations

*I*_K(DR)_	Delayed-rectifier K^+^ current
MDZ	Midazolam
IC_50_	Half maximal inhibitory concentration
IKCa	Intermediate-conductance Ca^2+^-activated K^+^ channel
DCEBIO	5,6-dichloro-1-ethyl-1,3-dihydro-2H-benzimidazol-2-one
FITC	Fluorescein isothiocyanate
PAP-1	PAP-1 (5-(4-Phenoxybutoxy)psoralen)
